# Metformin prevents hepatic steatosis by regulating the expression of adipose differentiation-related protein

**DOI:** 10.3892/ijmm.2013.1560

**Published:** 2013-11-19

**Authors:** FANG LIU, CHAO WANG, LIJUN ZHANG, YUQIAO XU, LINA JANG, YU GU, XIANGMEI CAO, XIN ZHAO, JING YE, QING LI

**Affiliations:** 1 State Key Laboratory of Cancer Biology, Department of Pathology, Xijing Hospital, Fourth Military Medical University, Xi’an, Shaanxi, P.R. China; 2 Department of Clinical Laboratory, Tangdu Hospital, Fourth Military Medical University, Xi’an, Shaanxi, P.R. China; 3 Department of Endocrinology and Metabolism, Xijing Hospital, Fourth Military Medical University, Xi’an, Shaanxi, P.R. China

**Keywords:** metformin, hepatic steatosis, lipids, PAT family, adipose differentiation-related protein

## Abstract

Non-alcoholic fatty liver disease (NAFLD) is a common liver disease, characterized by the excess accumulation of lipids in the liver. It has been demonstrated that the dysregulation of lipid droplet (LD)-associated proteins may be involved in the development of NAFLD. Adipose differentiation-related protein (ADRP), as one of the major LD-associated proteins, is expressed in normal and steatotic livers; however, the exact role of ADRP in the liver remains unknown. Previous studies have indicated that metformin, as an antidiabetic drug, effectively ameliorates NAFLD. However, its cellular and molecular mechanisms of action remain to be elucidated. Therefore, the aim of this study was to determine the role of ADRP in the metformin-mediated regulation of hepatic steatosis. We examined the effects of meformin *in vivo* and *in vitro* using *ob/ob* mice and primary hepatocytes, respectively. Lipid accumulation in the hepatocytes was induced by treatment with oleate. Our results revealed that metformin prevented hepatic steatosis in *ob/ob* mice and inhibited oleate-induced lipid accumulation in primary hepatocytes. Furthermore, using real-time PCR and western blot analysis, we examined the mRNA and protein expression of ADRP, respectively. We found that metformin significantly decreased the expression levels of ADRP. In addition, to further clarify the role of ADRP in lipid accumulation, we generated recombinant adenoviruses to induce the overexpression of ADRP and to knockdown ADRP. In the hepatocytes in which ADRP was overexpressed, the reducing effects of metformin on lipid accumulation were diminished. However, the knockdown of ADRP using siRNA targeting ADRP reduced the accumulation of triglycerides. Taken together, our data demonstrate that metformin prevents hepatic steatosis by regulating the expression of ADRP, which may be a key target in the treatment of NAFLD.

## Introduction

The global incidence of non-alcoholic fatty liver disease (NAFLD) continues to increase, and NAFLD is now recognized as a hepatic component of metabolic syndrome (1,2). In this regard, its occurrence is strongly associated with obesity, insulin resistance, hypertension and dyslipidemia (3). NAFLD covers a spectrum of hepatic pathologies, ranging from simple steatosis (fatty liver) to non-alcoholic steatohepatitis (4).

Metformin was introduced into clinical practice in the 1950s and is widely used as a first-line treatment for patients with type 2 diabetes (5). The effectiveness of metformin as an antidiabetic drug is explained by its ability to lower blood glucose levels by decreasing hepatic gluconeogenesis, stimulating glucose uptake into muscles, and increasing fatty acid oxidation in adipose tissue (6). It was previously demonstrated that some of the beneficial effects of this drug are related to an insulin-sensitizing effect through the activation of AMP-activated protein kinase (AMPK) (7). It is now accepted that the mitochondrial respiratory chain I is the primary target of metformin (8,9). Recent studies have reported that metformin is capable of ameliorating NAFLD (10); however, its exact mechanisms of action remain to be elucidated.

Hepatic steatosis refers to an excess accumulation of lipids, primarily triglycerides (TG), which is the hallmark of NAFLD. Lipid droplets (LDs) are spherical organelles composed of a core of neutral lipids covered by a monolayer of phospholipids and specific proteins. The excess accumulation of LDs in non-adipose tissues, such as the liver, coronary arteries or pancreatic islets, is often associated with fatty liver, coronary atherosclerotic heart disease and type 2 diabetes, respectively (11,12). Previous studies have identified LD-associated proteins that are involved in lipid metabolism and transport, intracellular trafficking, signaling and cytoskeletal organization (12–14). The most abundant and most well characterized family of LD-associated proteins is the PAT family, which is characterized by sequence similarity and its localization on LDs (15,16). The PAT family includes the following proteins: perilipin (PLIN)1, PLIN2 [also known as adipose differentiation related protein (ADRP)], PLIN3 [also known as tail-interacting protein 47 (TIP47)], PLIN4 (S3-12) and PLIN5 [also known as lipid storage droplet protein 5 (LSDP5) or OXPAT or MLDP].

It is well known that ADRP is the major LD-associated protein expressed in almost all cells, and it is widely used as a marker of LD. Previous *in vitro* studies have demonstrated that ADRP is involved in LD formation by enhancing the uptake of free fatty acids (FFA), thereby stabilizing LD particles (17,18). It has been demonstrated that the absence of ADRP expression reduces LD formation and protects against the development of fatty liver (19). TIP47 expression is present in the same cell types as ADRP (20) and can functionally compensate for it (21). However, the role of ADRP in liver diseases remains unknown.

In this study, in order to gain a better understanding of the role of ADRP in the alleviation of hepatic steatosis by metformin, we examined the effects of metformin in *ob/ob* mice and primary hepatocytes. Our results revealed that metformin prevented the development of hepatic steatosis by downregulating ADRP expression. These results suggest that ADRP may be a target of metformin in the treatment of NAFLD, which provides direct evidence of the mechanisms through which metformin inhibits hepatic lipid accumulation.

## Materials and methods

### Chemicals

Metformin (1,1-dimethylbiguanide hydrochloride) and the peroxisome proliferator-activated receptor (PPAR)α antagonist, GW6471, were purchased from Sigma-Aldrich (St. Louis, MO, USA). Anti-PPARα antibody was obtained from Cell Signaling Technology (Beverly, MA, USA), and the anti-α-tubulin antibody was purchased from Santa Cruz Biotechnology, Inc. (Santa Cruz, CA, USA). The Cy3-conjugated anti-mouse IgG secondary antibody was purchased from Invitrogen (Carlsbad, CA, USA). Collagenase type II was obtained from Sigma-Aldrich. Fatty acid-free bovine serum albumin (BSA) was purchased from Calbiochem (La Jolla, CA, USA). BODIPY 493/503 was also purchased from Invitrogen.

### Animal husbandry

Adult (aged 8–10 weeks) male *ob/ob* mice were generated by mating heterozygous leptin-deficient mice (*ob*/+) in a C57BL/6 background. Mice were housed in individual cages in a temperature-controlled environment with a 12-h light/dark cycle and were fed a standard laboratory chow diet. In this study, wild-type or *ob/ob* mice were randomly divided into 4 treatment groups as follows: group I (n=8), C57BL/6 mice were gavaged with distilled water; group II: C57BL/6 mice (n=8) were gavaged with metformin (75 mg/kg/day); group III: *ob/ob* mice (n=8) were gavaged with distilled water; and group IV: *ob/ob* mice (n=8) were gavaged with metformin (75 mg/kg/day). All mice were weighed at the beginning of the feeding period and weekly thereafter until the end of the experimental period, at which time tissues were collected for further analysis. All animal experiments were performed in accordance with the Guide for the Care and Use of Laboratory Animals of the National Institutes of Health and approved by the Ethics Committee of the Fourth Military University, Xi’an, China (Permit no: SCXK2007-007). All surgical procedures were performed under sodium pentobarbital anesthesia. We ensured that the animals did not suffer unnecessarily at any stage of this experiment.

### Isolation and culture of primary hepatocytes

Hepatocytes were isolated from male C57BL/6 mice by *in situ* digestion of the liver with perfusion of collagenase type II, as previously described (33). Following perfusion, the livers were immediately moved to a sterile 10 cm dish for mincing, before the hepatocytes were dispersed by aspiration with a large-bore pipette. The hepatocytes were then filtered through a 70-μm cell strainer (Millipore, Billerica, MA, USA) to remove tissue debris. After washing twice with cold DMEM and centrifuging at 50 × g for 3 min at 4ºC, an aliquot of freshly isolated hepatocytes was placed in a hemocytometer and stained with trypan blue to evaluate cell viability and determine the number of cells. Following isolation, 1×10^7^ cells were plated in 6-cm dishes and incubated at 37ºC in DMEM with 10% FBS, 10 μg·ml^−1^ penicillin and 100 μg·ml^−1^ streptomycin for 12–16 h. Prior to treatment with metformin, fresh DMEM was added to each dish; 1 h after the addition of metformin, the cells were treated with 200 μM oleate for 18 h. Duplicate dishes were used for all experiments. Metformin was used at a final concentration of 500 μM. A control solution containing ethanol and BSA was similarly prepared and administered.

### Generation and infection of recombinant adenovirus

The recombinant adenovirus expressing full-length ADRP (Ad-ADRP) was constructed using the AdEasy-1 System (Stratagene, La Jolla, CA, USA). The adenovirus carrying green fluorescent protein (GFP) (Benyuan Zhengyang Gene Technology Company Ltd., Beijing, China) was used as the control. We generated recombinant adenoviruses for the knockdown of ADRP (si-ADRP) and si-scramble as the control. Primary hepatocytes were infected with Ad-ADRP and Ad-GFP (control virus) or si-ADRP and si-scramble luciferase (si-control virus) in the presence or absence of metfromin, using Lipofectamine 2000 (Invitrogen) according to the manufacturer’s instructions. After 48 h, the cells were rinsed, collected and total RNA or protein was then extracted.

### Oil Red O staining

To detect fat accumulation in the liver and in primary hepatocytes, staining with Oil Red O was performed according to a previously described method (33). Briefly, liver cryosections and hepatocytes were fixed in 4% paraformaldehyde at room temperature for 30 min, and then dipped in 60% isopropanol for 3 min. After washing, the slides were immersed in freshly prepared 1% Oil Red O working solution for 15 min, and then washed in 60% isopropanol followed by distilled water, and finally mounted using aqueous mounting medium.

### Neutral lipid staining

The hepatocytes were seeded in a 12-well plate and treated with 500 μM metformin and 200 μM oleate for 18 h. After being washed twice with phosphate-buffered saline (PBS), the cells were fixed with 4% paraformaldehyde for 30 min at room temperature. BODIPY 493/503 (1 μg·ml^−1^) was used to stain the neutral lipid in the cells, and the nucleus was stained with Hoechst 33258 (10 μg·ml^−1^).

### Western blot analysis

Protein of the primary hepatocytes and liver was harvested using cold RIPA buffer (1% NP-40, 50 mM Tris-HCl, 0.1% SDS, 1% sodium deoxycholate, 150 mM NaCl, pH 7.4), containing leupeptin (2 μg·ml^−1^) and sodium orthovanadate (2 μg·ml^−1^). All mixtures were then centrifuged at 12,000 × g at 4ºC for 10 min, and the protein content of the supernatant was determined using a commercially available kit (Bio-Rad Laboratories, Hercules, CA,USA), using BSA as the standard.

Equal amounts of protein samples were resolved by SDS-PAGE, and then transferred onto PVDF membranes (Millipore). The membranes were immunoblotted with antibodies against PLIN (Abcam, Cambridge, UK), ADRP, (Abcam) and α-tubulin (Santa Cruz Biotechnology, Inc.). Antibodies generated in our own laboratory were used for LSDP5 (monoclonal antibody) and TIP47 (polyclonal antibody).

### Real-time PCR

Total RNA was extracted from the liver samples using TRIzol reagent (Invitrogen) and cDNA produced using a reverse transcription kit (Takara, Dalian, China). Quantitative analysis of the mRNA levels was performed by real-time PCR using a Master Mix SYBR-Green kit (Takara), according to the manufacturer’s instructions. The primer sequences for all genes of interest are listed in Table I. GAPDH was used as a reference gene in all experiments. Relative abundance of RNA was calculated from the cycle threshold (Ct) using the 2^−ΔΔCt^ method and expressed in arbitrary units.

### Blood sampling and biochemical assays

Blood samples were obtained after 4 weeks of treatment with metformin and stored at 4ºC until use in biochemical analysis. Serum total cholesterol, TG and blood glucose, as well as high-density lipoprotein (HDL) and low-density lipoprotein (LDL) cholesterol levels were measured using automated techniques within the Department of Clinical Chemistry, the Fourth Military Medical University.

### Lipid extraction and thin layer chromatography (TLC)

To measure the total TG levels, lipids were extracted from the tissues and cells using the Folch method, as previously described (30). In brief, the dried lipids were reconstituted in chloroform/methanol (2:1, v/v) and loaded onto a TLC plate (Sigma-Aldrich). The lipids were separated in a hexane/diethyl ether/acetic acid (70:30:1, v/v/v) solution. The TLC plates were sprayed with 10% CuSO_4_ in 10% phosphoric acid and were developed by drying in a 120ºC oven. The protein concentration was determined using the Bio-Rad Protein Assay (Bio-Rad #500-0001) and the amount of TG was quantified using Bio-Rad Quantity One Software (Bio-Rad Laboratories).

### Statistical analysis

All data are expressed as the means ± standard error of the mean (SEM), and analyzed using a paired sample two-sided Student’s t-test for paired samples or one-way ANOVA and Dunnett’s post-test using SPSS version 13.0 (SPSS Inc., Chicago, IL, USA). A value of P<0.05 was considered to indicate a statistically significant difference.

## Results

### Metformin prevents the development of fatty liver in ob/ob mice and inhibits lipid accumulation in primary hepatocytes

**Figure 1:**
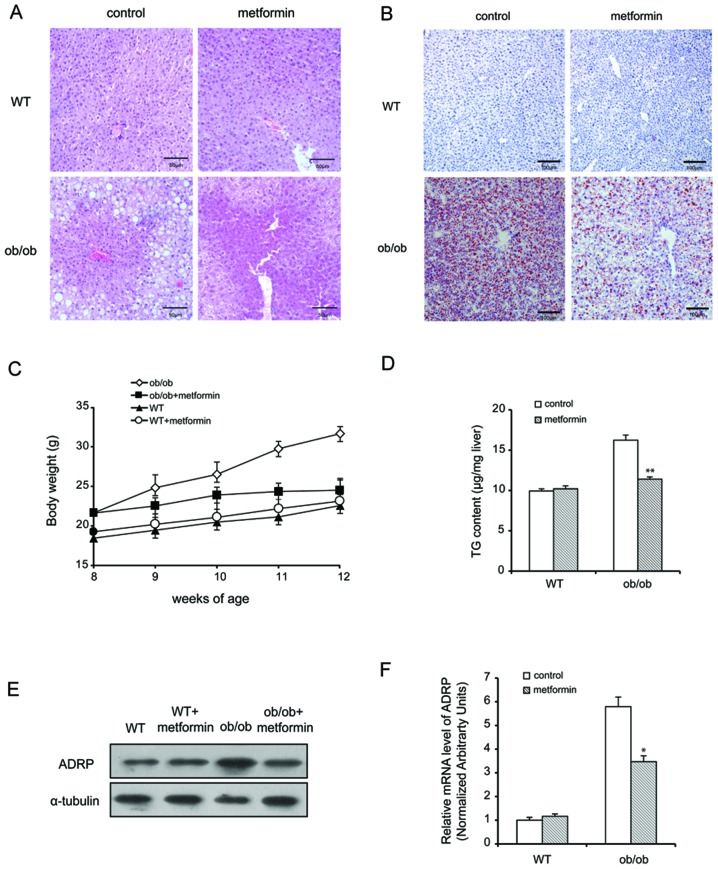
Metformin alleviates hepatic steatosis in *ob/ob* mice. (A) Hematoxylin and eosin (H&E) staining of liver sections from wild-type (WT; C57BL/6, control) or *ob/ob* mice treated without or with metformin for 4 weeks (scale bar, 50 μm). (B) Representative frozen tissue sections of livers stained with Oil Red O to demonstrate the reduction in lipid droplets (scale bar, 100 μm). (C) Body weight of the experimental mice is shown. (D) Hepatic triglyceride (TG) concentration was quantified using thin layer chromatography. All data are expressed as the means ± SEM (n=8) for each group. ^**^P<0.05. (E) Hepatic adipose differentiation-related protein (ADRP) protein level was detected by western blot analysis, α-tubulin was used as an internal control for equal loading of the samples. (F) Hepatic ADRP mRNA expression was assessed by real-time PCR. Data shown are the means ± SEM of representative experiments performed in triplicate. ^*^P<0.05.

To investigate the potential role of metformin in the prevention of hepatic steatosis, we used *ob/ob* mice presenting with hepatomegaly, as previously described (22). After 4 weeks of metformin treatment (75 mg/kg/day), the body weight, serum TG, LDL cholesterol and glucose levels in the *ob/ob* mice decreased significantly; however, there was no change in the total cholesterol and HDL cholesterol levels, compared with the control groups (Table II). Hematoxylin and eosin (H&E) and Oil Red O staining demonstrated that treatment with metformin reduced the number and size of LDs in the hepatocytes treated with oleate, and ameliorated fatty liver in the *ob/ob* mice (Fig. 1A and B). Of note, the metformin-treated *ob/ob* mice failed to gain additional weight at the beginning or end of the experimental period, although both groups of C57BL/6 (wild-type) mice gained weight (Fig. 1C). Lipid extraction and TLC revealed that the levels of hepatic TG significantly decreased in the metformin-treated mice (Fig. 1D), supporting our histological results. To investigate the potential molecular mechanisms underlying the inhibitory effects of metformin on the development of liver steatosis, we first explored the effects of metformin on hepatic ADRP expression by western blot analysis and real-time PCR. As illustrated in Fig. 1E and F, following treatment with oleate, the mRNA and protein levels of hepatic ADRP were increased by almost 6-fold; however, treatment with metformin significantly reduced the levels of ADRP by 43% compared with the control group (P<0.05).

**Figure 2:**
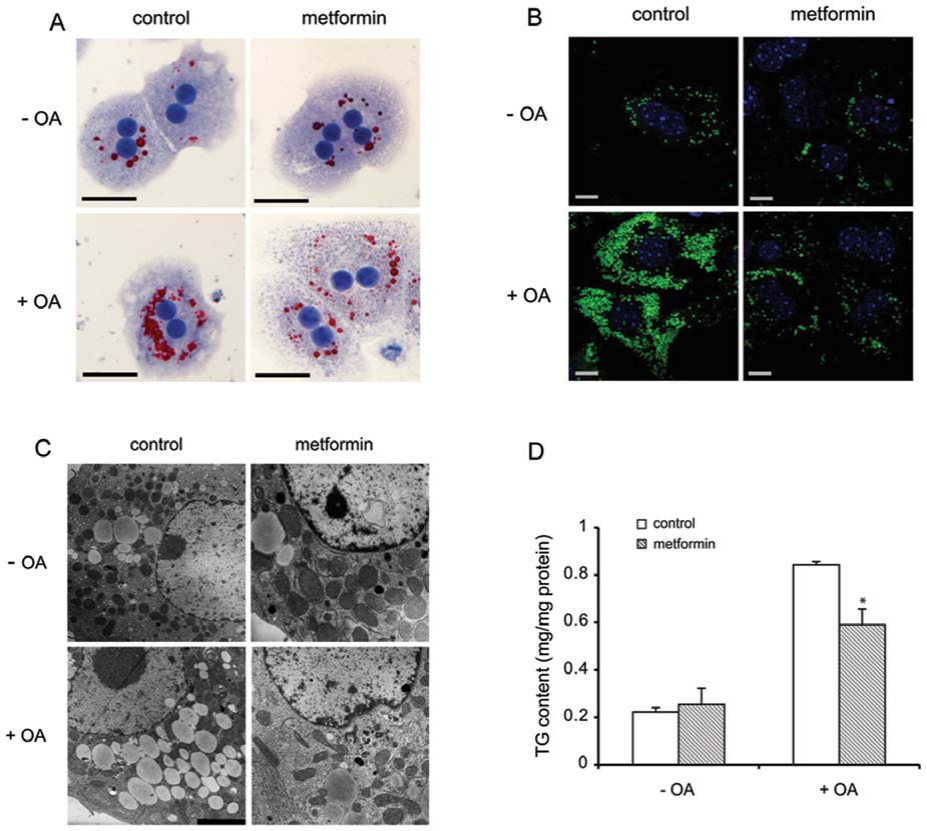
Metformin diminishes lipid accumulation in primary hepatocytes. (A) Primary hepatocytes were stained with Oil Red O (scale bar, 20 μm). Primary hepatocytes pre-incubated without or with 500 μM metformin for 12 h, then stimulated without or with 200 μM oleate for 18 h. (B) The lipid droplets in primary hepatocytes were visualized using BODIPY 493/503 (green staining). Nuclei were stained with Hoechst 33258 (blue staining) (scale bar, 15 μm). (C) Electron microscopic analysis was used to assess lipid droplet morphology and number (scale bar, 2 μm). (D) The cellular concentration of triglycerides (TG) was quantified using thin layer chromatography. All data are expressed as the means ± SEM (n=4) for each group. ^*^P<0.05.

To further evaluate the physiological role of metformin in the prevention of hepatic steatosis, we employed primary hepatocytes to confirm our *in vivo* results using an *in vitro* model. Oil Red O, BODIPY 493/503 staining and electron microscopic analysis revealed that lipid loading significantly decreased in the metformin-treated hepatocytes, compared with the control cells (Fig. 2A–C). Furthermore, metformin markedly reduced the TG content by 33% in the primary hepatocytes, as analyzed by TLC (Fig. 2D).

### Metformin suppresses the expression of ADRP

**Figure 3:**
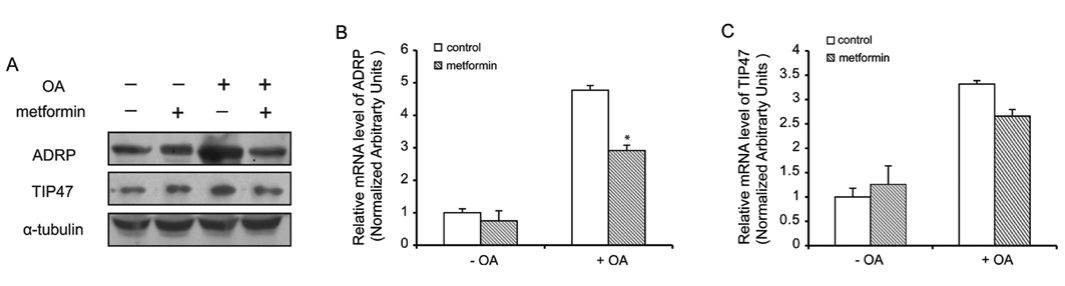
Metformin decreases the expression of adipose differentiation-related protein (ADRP) in primary hepatocytes. (A) Isolated primary hepatocytes were pre-treated with 500 μm metformin and then incubated with 200 μm oleate for 18 h. Representative western blots showing the expression level of ADRP and tail-interacting protein 47 (TIP47) proteins in primary hepatocytes. α-tubulin was used as an internal control for the equal loading of samples. Representative blots shown were from 3 independent experiments with identical results. (B and C) Relative mRNA levels of ADRP and TIP47 are also shown. The relative mRNA level in the control group was designated as 1.0. n=4, ^*^P<0.05.

The function of LDs is regulated by their associated proteins. To confirm whether the effects of metformin are a result of a decrease in the expression of PAT family proteins, we examined PAT family protein levels in hepatocytes treated with metformin. Our western blot analysis data demonstrated that ADRP and TIP47 protein levels were markedly decreased in the oleate-treated hepatocytes pre-treated with metformin, compared with the control cells (Fig. 3A). However, metformin did not inhibit the expression of PLIN, S3-12 and LSDP5 (data not shown). As expected, and as shown in Fig. 3B, metformin decreased the mRNA level of ADRP by almost 40%, but failed to decrease the expression of TIP47 (Fig. 3C) and other PAT proteins (data not shown).

### Induction of ADRP overexpression by Ad-ADRP diminishes the reducing effects of metformin on TG accumulation

**Figure 4:**
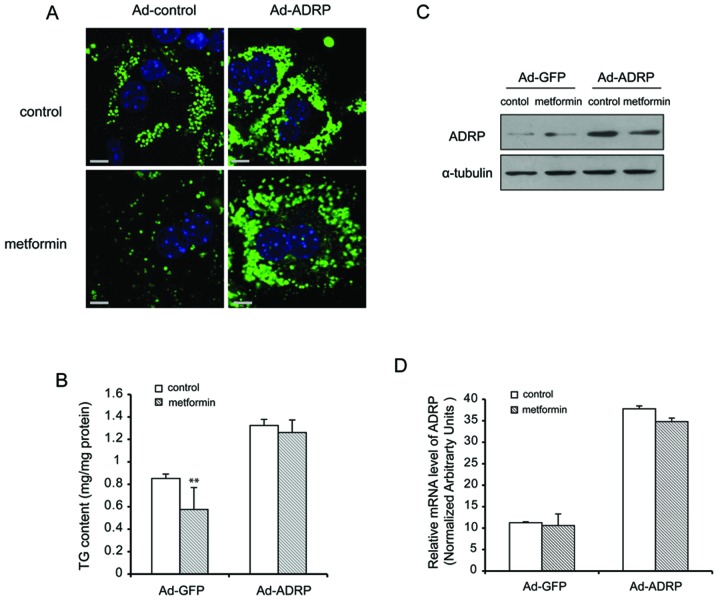
Pre-treatment with recombinant adenovirus expressing adipose differentiation-related protein (ADRP) promotes triglyceride (TG) accumulation. Primary mouse hepatocytes were infected with Ad-ADRP or Ad-GFP (control) for 6 h and then incubated with 200 μM oleate and 500 μM metformin for 24 h. (A) The lipid droplets in primary hepatocytes were visualized using BODIPY 493/503 (green staining). Nuclei were stained with Hoechst 33258 (blue staining) (scale bar, 15 μm). (B) A higher TG content was observed in primary mouse hepatocytes overexpressing ADRP treated with metformin compared with the control. (C) ADRP protein level was detected by western blot analysis; α-tubulin was used as the control. Data are presented as the means ± SEM (n=4), ^**^P<0.01. (D) The relative mRNA level of ADRP is also shown. The relative mRNA level in the control group was designated as 1.0. n=4.

To determine whether the induction of ADRP overexpression plays an indispensable role in the metformin-mediated storage of TG, primary hepatocytes were infected with adenovirus expressing either ADRP or GFP. As shown in Fig. 4A, LDs were stained using BODIPY. BODIPY fluorescence increased in the cells infected with Ad-ADRP; however, the fluorescence signal did not decrease in the presence of metformin. As shown in Fig. 2B, the overexpression of ADRP increased the cellular TG levels by 40% compared with the Ad-GFP-infected (control adenovirus) hepatocytes. However, following treatment with metformin, the amount of TG did not decrease to the levels of the control. The expression of ADRP was measured by western blot analysis and real-time PCR (Fig. 4C and D). The results revealed that after the cells were infected with Ad- ADRP, the expression of ADRP increased by almost 3.7-fold.

### Metformin-mediated lipid accumulation is enhanced by the absence of ADRP in primary hepatocytes

**Figure 5:**
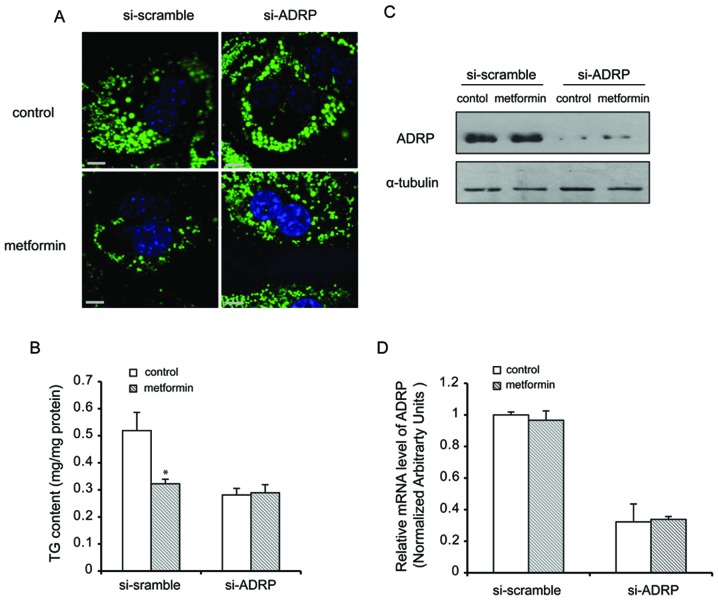
Effect of treatment with siRNA targeting adipose differentiation-related protein (ADRP) lipid accumulation in primary hepatocytes. Primary hepatocytes were infected with an adenovirus carrying ADRP siRNA for 6 h and then incubated with 200 μM oleate and 500 μM metformin for 24 h. (A) BODIPY staining of cells infected with ADRP siRNA treated with metformin (green staining). Nuclei were stained with Hoechst 33258 (blue staining) (scale bar, 15 μm). (B) Concentration of triglycerides (TG) was determined in si-ADRP-infected cells treated with metformin, compared with the si-scramble (control)-infected cells. Data are presented as the means ± SEM (n=4), ^*^P<0.05. (C) Western blot analysis revealed that the expression level ADRP in the cells infected with si-ADRP or si-scramble and treated with metformin; α-tubulin was used as the control. (D) The relative mRNA level of ADRP is also shown. The relative mRNA level in the control group was designated as 1.0. n=4.

To further deterime the effects of metformin on lipid metabolism, we used an adenovirus-mediated gene silencing approach. ADRP mRNA was successfully reduced below 70% by si-ADRP compared with si-scramble (control). Following pre-treatment with si-ADRP and treatment with metformin, the relative intracellular lipid content in each group was determined. The results (Fig. 5A) revealed that BODIPY fluorescence in the cells infected with si-ADRP was less than that in the cells infected with si-scramble (control) under lipid loading conditions, suggesting that si-ADRP prevented lipid accumulation with or without metformin treatment. Pre-treatment with si-ADRP markedly reduced the TG content in the cells by 37% when compared with the control group (P<0.05) (Fig. 5B). Western blot analysis of the cells treated with metformin and pre-treated with si-ADRP revealed the reduced protein and mRNA expression of ADRP (Fig. 5C and D).

Collectively, the data from overexpression and silencing experiments indicated that ADRP may repesent a key and direct cellular target in the metformin treatment of hepatic steatosis.

## Discussion

It has previously been reported that metformin, a commonly used drug for the treatment of diabetes, is capable of ameliorating NAFLD (23,24); however, its exact mechanisms of action remain to be elucidated. The hypothesis that respiratory chain complex I, but not AMPK, is the primary target of metformin was recently strengthened by a study demonstrating that the metabolic effects of this drug are preserved in liver-specific AMPK-deficient mice (25), although the implications of this result with respect to NAFLD remain to be elucidated. In this study, we explored the physiological functions of metformin in the improvement of hepatic steatosis and provide a direct mechanistic understanding of the mechanisms through which metformin alleviates hepatic lipid accumulation. In agreement with previous studies, we demonstrated that metformin prevented lipid accumulation and hepatic steatosis in *ob/ob* mice, and prevented lipid accumulation in isolated primary hepatocytes. Furthermore, we observed that the number and size of LDs and the TG content markedly decreased following treatment with metformin *in vivo* and *in vitro*.

Although an increasing number of studies have indicated that metformin activates AMPK, the direct role of metformin in the regulation of hepatic lipid accumulation remains to be clarified. PAT family proteins are major regulators of various aspects of lipid metabolism, including the promotion of lipid storage (26) and the recruitment of lipases or co-lipases (19,27). ADRP is the major LD protein in all cells that accumulate lipids either normally or abnormally. ADRP is considered a reliable and sensitive marker for LDs in steatosis, and it promotes the incorporation of lipids in LDs at the cost of reduced lipid export via lipoprotein secretion or reverse transport (20); by contrast, TIP47 preferentially labels nascent LDs (28) and binds to the LDs in response to lipid loading (19). ADRP binding to LDs is ubiquitous in non-adipose LD-containing cells (20,29), and plays important roles in LD formation and stabilization, as well as lipolysis (30). In the liver, ADRP is upregulated in association with drug- and diet-induced hepatic steatogenesis (28,31). Reduced ADRP expression either in ADRP-deficient mice (19), or in mice administered ADRP antisense oligonucleotides while on a high fat diet (32), attenuates hepatic steatosis. To elucidate the mechanisms of action of metformin in the improvement of hepatic steatosis, our study provides novel insight into the association between metformin and PAT family proteins. Our data demonstrated that the expression of ADRP was predominant out of the PAT family proteins, and was markedly decreased following treatment with metformin in the livers of mice and primary hepatocytes. Thus, we focused on ADRP, which seems to play the most singificant role in hepatic steatosis, and the effects of metformin on its expression.

Our study provides evidence that ADRP plays a crucial role in the prevention of hepatic steatosis by metformin. ADRP expression was increased in hepatocytes treated with oleate and decreased following treatment with metformin. Transfection with Ad-ADRP increased the storage of TG and si-ADRP prevented lipid accumulation. Of note, the reducing effects of metformin on TG accumulation were diminished in the hepatocytes infected with Ad-ADRP. Most importantly, to our knowledge, we identified for the first time that metformin treatment decreases the expression of ADRP. These findings suggest that ADRP mediates the prevention of hepatic steatosis by metformin.

Overall, our data demonstrate that metformin prevents hepatic steatosis *in vivo* and *in vitro* by inhibiting the expression of LD-associated proteins, namely ADRP. The downregulation of ADRP enhanced the oxidation of fatty acids and reduced lipid synthesis, thus preventing lipid accumulation in the hepatocytes, contributing to the prevention of hepatic steatosis. In conclusion, our study demosntrates that metformin exerts an inhibitory effect on ADRP expression; thus, it may be useful to further establish ADRP as a marker for liver steatosis. Our data provide further insight into the possible mechanisms underlying the improvement of liver steatosis following tretment with metformin.

## Figures and Tables

**Table I:** Sequences of primers used for real-time PCR.

Gene	Sequence (5′→3′)
GAPDH	F: AACCCCTTCATTGACCTCAACTAC R: ATTTGATGTTAGTGGGGTCTCGCT
ADRP	F: GATTGAATTCGCCAGGAAGA R: TGGCATGTAGTCTGGAGCTG
TIP47	F: GTGTGGGACAGATGGTGAT R: AAGTAGTTCTGCTCCTGTCG

ADRP, adipose differentiation-related protein; TIP47, tail-interacting protein 47; F, forward; R, reverse.

**Table II:** Effects of metformin on serum biochemistry (mM).

Biochemical markers (mM)	Group I	Group II	Group III	Group IV
Glucose	6.76±0.75	6.51±0.6	11.11±1.54^b^	5.99±0.13^d^
Triglycerides	0.55±0.05	0.57±0.06	0.88±0.08^b^	0.6±0.11^d^
Cholesterol	1.62±0.07	1.67±0.14	2.12±0.27^b^	2.08±0.34
HDL	1.41±0.43	1.33±0.34	2.03±0.28^a^	2.41±0.54
LDL	0.25±0.06	0.26±0.04	0.33±0.07^a^	0.29±0.04^c^

Sixteen male C57BL-6 mice and 16 male *ob/ob* mice were divided into 4 groups. Serum concentrations of glucose, triglycerides, cholesterol, high-density lipoprotein (HDL) and low-density lipoprotein (LDL) were measured in the C57BL-6 mice (group I), C57BL-6 mice treated with metformin (group II), *ob/ob* mice (group III) and *ob/ob* mice treated with metformin (group IV). Group I vs. group III: ^a^P<0.01, ^b^P<0.001; Group III vs. group IV: ^c^P<0.05, ^d^P<0.001.
